# Redefining Roles—Fathers Play a Crucial Role in Shaping Children’s Healthy Eating Behaviors: Cross-Sectional Observational Study

**DOI:** 10.3390/nu17050860

**Published:** 2025-02-28

**Authors:** Nicholas Beng Hui Ng, Jamie Qiao Xin Ng, Liang Shen, Shefaly Shorey

**Affiliations:** 1 Department of Paediatrics, Khoo Teck Puat-National University Children’s Medical Institute, National University Health Systems, Singapore 119074, Singapore; 2 Department of Paediatrics, Yong Loo Lin School of Medicine, National University of Singapore, Singapore 117597, Singapore; 3 Alice Lee Centre for Nursing Studies, Yong Loo Lin School of Medicine, National University of Singapore, Singapore 117599, Singapore; 4 Biostatistics Unit, Yong Loo Lin School of Medicine, National University of Singapore, Singapore 117597, Singapore

**Keywords:** responsive feeding, fathers, child feeding practices, child eating habits

## Abstract

Background/Objectives: Fathers have been largely understudied in feeding research, as most studies have focused on mothers. This study aims to explore the relationship between paternal feeding practices and child eating behaviors. Methods: This study employed a cross-sectional observational design. Fathers (*n* = 114) completed one-off self-reported questionnaires using the Child Feeding Practices Questionnaire and the Child Eating Behaviors Questionnaire from October 2023 to February 2024. Variations in feeding practices across sociodemographic factors were explored using correlational statistics. The generalized linear model was used to identify relationships between paternal feeding practices and child eating behaviors. Results: For young children, the availability of healthy food at home was positively associated with food approach behaviors, while paternal practices of pressuring and child control were linked to increased food avoidance. Among school-aged children, using food as a reward was positively associated with food approach behaviors, while using food to regulate emotions was linked to increased food avoidance. Restriction for weight control was associated with both food approach and avoidant behaviors. In adolescents, paternal monitoring of their diet and certain socioeconomic conditions were observed to be associated with their eating behaviors. Conclusions: The findings provide valuable insights into the role of paternal feeding practices in shaping children’s eating behaviors and highlight the potential for interventions targeting modifiable paternal behaviors to support healthy eating habits.

## 1. Introduction

Healthy eating behaviors are essential for the growth, development, and long-term health outcomes of children [1,2]. However, the global rise in childhood obesity has become a significant global health concern [3]. For instance, Singapore experienced increasing childhood obesity rates, from 13% in 2017 to 16% in 2021 [4,5], contributed from sedentary lifestyles, increased consumption of energy-dense foods, and cultural shifts towards Westernized diets [6]. Childhood obesity is strongly associated with adult obesity [7,8], with increased risks of metabolic co-morbidities, such as cardiovascular diseases and diabetes [9]. Children eating behaviors influence their risk of developing obesity [10], with excessive food approach being significantly associated with childhood obesity [11]. Conversely, food avoidance behaviors are inversely associated with childhood obesity [12,13], though excessive food-avoidant behaviors may lead to restrictive food intake disorders [14,15].

Responsive feeding, where caregivers recognize and feed their child in response to the child’s hunger and satiety cues, has been shown to foster self-regulation and develop healthy eating behaviors [16], reducing the risk of childhood obesity [17]. Parental feeding practices are key in shaping dietary behaviors and nutritional outcomes in children [18], but most research has centered on mothers [19,20,21]. Although fathers are increasingly involved in childcare, they remain under-represented in the current literature [22,23], despite evidence suggesting their significant influence on long-term eating behaviors in their children [24]. Existing studies have been largely conducted in Western countries [25,26,27], which may not be entirely generalizable to Singapore [28]. Despite being a small nation, Singapore has a unique composition, with multiracial and diverse religious composition. In fact, individuals from different ethnic backgrounds within Singapore have been found to differ in parenting styles, possibly influencing local eating practices in children [29]. These reasons necessitate an up-to-date examination of fathers’ feeding practices and their children’s eating behaviors in a multicultural Asian context like Singapore. Hence, this cross-sectional study aims to explore paternal feeding practices and their association with child eating behaviors.

## 2. Materials and Methods

### 2.1. Study Design and Participants

This study used a cross-sectional study design and represented the quantitative component of a broader sequential mixed-method study focusing on Singaporean feeding practices. This study was conducted from October 2023 to February 2024. The reporting of this study adhered to the STrengthening the Reporting of OBservational studies in Epidemiology (STROBE) reporting guidelines (Table S1). A convenient sample of fathers was recruited from a general pediatric outpatient clinic in Singapore. Fathers were included if they were (1) between 21 and 65 years of age (legal age of consent in Singapore), (2) had a child whose age fell into one of three age groups (young children: 0–6 years old; school-aged children: 7–12 years old; and adolescents: 13–18 years old), and (3) were able to read, speak, and comprehend English. Fathers with any physical or mental disabilities that hindered their ability to complete the questionnaires were excluded from this study.

### 2.2. Sample Size Calculation

Power analysis for multiple linear regression was used to calculate the sample size required for this study. As 10 variables, including sociodemographic and outcome variables, were considered in the linear regression model, a minimum of 95 participants were required to achieve a medium effect size (f^2^ = 0.15) with 80% power and a significance level of 0.05 (2-tailed) [30]. Factoring in an attrition rate of 20% based on previous local studies with fathers, 114 fathers were recruited in this study [31,32].

### 2.3. Data Collection

Data were collected in English using self-reported questionnaires on a secure and ethically approved online platform called Qualtrics [33].

The questionnaires used in this study were the Child Feeding Practices Questionnaire and the Child Eating Behaviors Questionnaire, which had been previously validated in a cohort of Singaporean children [12,13,34,35]. Cronbach’s alpha was used to assess the internal consistency of each instrument in this study. Demographic information, such as age, BMI, ethnicity, highest educational background of both the father and their partner, current employment status, occupation, working hours, monthly household income, number of children, and child’s age, sex, and BMI, was collected.

#### 2.3.1. Child Feeding Practices Questionnaire

Fathers’ feeding practices were measured using the Child Feeding Practices Questionnaire (CFPQ) [18]. The questionnaire consisted of 49 items related to 12 factors of various parental feeding practices: (1) child control (5 items, Cronbach’s α = 0.678); (2) emotional regulation (3 items, Cronbach’s α = 0.673); (3) encourage balance and variety (4 items, Cronbach’s α = 0.345); (4) environment (4 items, Cronbach’s α = 0.608); (5) food as reward (3 items, Cronbach’s α =0.672); (6) involvement (3 items, Cronbach’s α = 0.559); (7) modeling (4 items, Cronbach’s α = 0.832); (8) monitoring (4 items, Cronbach’s α = 0.884); (9) pressure to eat (4 items, Cronbach’s α = 0.674); (10) restriction for health (4 items, Cronbach’s α = 0.631); (11) restriction for weight control (8 items, Cronbach’s α = 0.822); and (12) teaching about nutrition (3 items, Cronbach’s α = 0.563). Most of the factors in this study had acceptable scores for internal consistency, as values near 0.6 could be considered acceptable for factors with a small number of items [36]. However, subscales with particularly low Cronbach’s alpha values (<0.5), such as (3) encourage balance and variety, were removed from the analysis to ensure the reliability of the findings. Previous studies using the CFPQ have also reported varying internal consistency values for these subscales, with some studies also observing lower Cronbach’s alpha scores [37,38]. For subscales with alpha values between 0.5 and 0.6 (i.e., involvement and teaching about nutrition), they were retained to preserve the breadth of parental feeding practices assessed in this study. Higher scores meant that fathers were more likely to use that specific feeding practice. The questionnaire was also locally validated among mothers of 5-year-old children (Cronbach’s α = 0.56 to 0.86) and parents of children between 3 and 6 years of age (Cronbach’s α = 0.54 to 0.80).

#### 2.3.2. Child Eating Behaviors Questionnaire

Children’s eating behaviors were measured using the Child Eating Behaviors Questionnaire (CEBQ) [10]. The questionnaire consisted of 35 items measuring 8 factors that could be divided into 2 dimensions: food approach and food avoidance. The food approach consisted of 4 factors: food responsiveness (5 items, Cronbach’s α = 0.70), food enjoyment (4 items, Cronbach’s α = 0.82), emotional overeating (4 items, Cronbach’s α = 0.77), and desire to drink (3 items, Cronbach’s α = 0.72). Food avoidance consisted of 4 factors: satiety responsiveness (5 items, Cronbach’s α = 0.72), slowness in eating (4 items, Cronbach’s α = 0.84), food fussiness (6 items, Cronbach’s α = 0.89), and emotional undereating (4 items, Cronbach’s α = 0.76). All items were rated on a 5-point Likert scale: 1 (never), 2 (rarely), 3 (sometimes), 4 (mostly), and 5 (always). The questionnaire was also locally validated in studies involving parents of 3-, 5-, and 6-year-old children (Cronbach’s α = 0.70 to 0.88).

#### 2.3.3. Anthropometric Measures

Fathers reported the height and weight of their children immediately after obtaining measurements taken by the clinic’s staff, just before the doctor’s appointment. The height and weight measurements for the children were obtained by trained pediatric ambulatory nursing staff using a calibrated weighing scale (SECA-803) and a stadiometer (SECA-213). The height and weight measurements were then used to calculate the BMI standardized score or BMI-z (BAZ), which refers to the BMI adjusted for the child’s respective age and gender using the WHO Anthro software (Version 3.2.2) [39].

### 2.4. Data Analysis

All statistical analyses were conducted using the Statistical Package for the Social Sciences (SPSS) version 29.0. Frequencies and percentages were used to describe categorical data, while mean and standard deviations (SDs) were used to describe continuous data. Kruskal–Wallis tests were used to examine variations in paternal feeding practices across categorical variables, such as ethnicity, education levels, employment statuses, income levels, the number of children in the household, and a child’s sex. Spearman’s correlation was used to examine relationships between feeding practices across numerical variables, including the father’s age, BMI, working hours, and the child’s age and BAZ.

Due to the cognitive, social, and emotional changes experienced during differing stages of development, the sample population was split into three age groups, (1) young children (0 to 6 years old), (2) school-aged children (7 to 12 years old), and (3) adolescents (13 to 18 years old), for analysis. Bivariate regression models of the twelve separate feeding practice domains (e.g., child control and emotional regulation) were used to examine the effect of feeding practices on child food approach and food avoidance behaviors. After confirming that there were no signs of multicollinearity across the domains of feeding practices, the final multivariate generalized linear models were used to report the results of this study. If no statistically significant factor was identified by any multivariate model, an additional model consisting of variables that had *p* < 0.10 in their univariate models was built.

### 2.5. Ethical Considerations

The National Healthcare Group domain-specific review board, which is the ethics board of the participating hospital, approved this study on 24 August 2023, before the commencement of recruitment (NHG2023/00461). Written informed consent was obtained from the fathers, and voluntary participation was reinforced. Anonymity was maintained as each father was assigned a unique participant code during this study.

## 3. Results

### 3.1. Demographics

A total of 114 fathers were recruited for this study. The mean age of the fathers was 44.9 years of age (SD = 6.17), and the majority of the fathers were Singaporean Chinese (70.2%, 90/114). Most of the fathers had at least a university degree (76.3%, 87/114) and were employed full-time (86%, 98/114), with a monthly household income of more than SGD 10,000 (Singapore Dollars) (60.5%, 69/114). Fathers worked an average of 8.86 h (SD = 1.55). Most fathers had two children (60.5%, 69/114) in their family. The mean age of the children who visited the clinic was 8.64 years of age (SD = 4.28), and their median BAZ score was −0.10 (SD = 1.56). More details are presented in Table 1.

### 3.2. Paternal Feeding Practices

The most widely adopted feeding practice among Singaporean fathers was teaching about nutrition using didactic methods across all age groups. The least adopted feeding practice was using food to regulate their children’s emotional status across all age groups (Table S2).

Bivariate regression models were used to examine the variations in paternal feeding practices across sociodemographic factors, and the results are presented in Tables S3 and S4.

### 3.3. Children Eating Behaviors

Results from the general linear model identified two factors that were statistically significantly associated with food approach behaviors in young children between 0 and 6 years of age (Table 2). Availability of healthy foods at home (environment, β = 1.49, *p* = 0.07) was statistically significantly associated with greater food approach behaviors in young children. Fathers’ working hours (β = −1.00, *p* = 0.02) were negatively associated with food approach behaviors in young children.

Two factors were found to be significantly associated with food-avoidant behaviors in young children between 0 and 6 years of age (Table 2). Fathers pressuring children to eat during mealtimes (pressure to eat; β = 2.03, *p* < 0.001) and encouraging child autonomy in eating (child control; β = 0.733, *p* = 0.032) were significantly associated with greater food avoidance behaviors. The gender of the child was not significantly associated with food avoidance behaviors in the final generalized linear model.

Two factors were significantly associated with food approach behaviors in school-aged children between 7 and 12 years old (Table 3). Fathers’ restriction of their child’s intake for weight control (restriction for weight control, β = 0.39, *p* = 0.012) and use of food to reward positive behaviors (food as reward, β = 0.73, *p* = 0.042) were significantly associated with greater food approach behaviors in school-aged children. Fathers’ overt monitoring of their child’s intake and their child’s BMI z-score was not significantly associated with food approach behaviors in the final model.

Two factors were found to be significantly associated with food-avoidant behaviors in school-aged children between 7 and 12 years old (Table 3). Fathers use of food to regulate their children’s emotions (emotional regulation; β = 2.14, *p* = 0.008) was positively associated with food-avoidant behaviors while restricting their child’s intake for weight control (restriction for weight control; β = −0.56, *p* = 0.013) was negatively associated with food avoidance.

The general linear model revealed that five factors were significantly associated with food approach behaviors in adolescents between 13 and 18 years old (Table 4). Paternal monitoring of adolescents’ diets (monitoring; β = 0.99, *p* = 0.002) was positively associated with food approach behaviors. Adolescents with Chinese fathers reported higher appetitive behaviors compared to adolescents with fathers from non-Chinese ethnicity (β = 5.70, *p* = 0.032). Adolescents growing up in households with one child (β = 8.31, *p* = 0.032) and two children (β = 5.81, *p* = 0.019) reported significantly higher appetitive behaviors compared to households with three or more children. Adolescents growing up in less affluent households with a monthly household income of less than SGD 6000 (equivalent to less than USD 4500) also reported significantly higher appetitive behaviors when compared to higher-income households (β = 5.91, *p* = 0.022).

Three types of paternal feeding practices were significantly associated with food-avoidant behaviors in 13–18-year-old adolescents. Fathers’ use of food to regulate their adolescents’ emotions (emotional regulation; β = 1.95, *p* = 0.048) and restriction of their adolescents’ diet to limit unhealthy food intake (restriction for health; β = 1.90, *p* < 0.001) were positively associated with food-avoidant behaviors. Fathers who were employed full-time reported statistically significantly lower food-avoidant behaviors in their adolescents, compared to fathers who were not employed full-time (β = −8.29, *p* = 0.010).

## 4. Discussion

This study aimed to explore paternal feeding practices and their associations with child eating behaviors. Among young children, the availability of healthy food at home was associated with food approach behaviors, whereas fathers’ working hours showed a negative association. Paternal pressure to eat and encouragement of child autonomy were associated with food avoidance in this age group. Among school-aged children, using food as a reward and restricting food for weight control were connected to food approach behaviors. Additionally, using food for emotional regulation and restricting food for weight control were related to food avoidance in school-aged children. Adolescents from low-income, two-child, or Chinese households, as well as those whose fathers monitored their diets, exhibited more food approach behaviors. The use of food to regulate their adolescents’ emotions and restriction for health were positively associated with food avoidance among adolescents. In contrast, paternal full-time employment were negatively associated with food avoidance among adolescents.

Our findings show that most fathers in Singapore primarily used responsive rather than non-responsive feeding practices. In contrast, other studies have reported that most American and British fathers adopted non-responsive feeding practices such as pressuring their children to eat more and restricting their child’s food intake to control their child’s weight [40,41,42]. This could be attributed to the comprehensive and extensive national healthy eating campaigns that may have raised awareness of responsive feeding practices in Singapore [43].

Availability of healthy food at home was positively associated with food approach behaviors, while fathers’ working hours were negatively associated with food approach behaviors among young children. Previous studies also reported that the availability of healthy foods at home, such as fruits and vegetables, was associated with a higher intake of these healthy foods by younger children due to early and frequent exposure to healthy food [44,45]. Another local study found that working mothers often had limited influence over their children’s meal intake, as domestic workers, childcare centers, and other proxy caregivers were in charge of meal provision [46]. Fathers who work long hours likely have fewer opportunities to prepare or participate in family meals, reducing their influence on their children’s eating habits. As a result, they may observe and report lower food approach behaviors in their children. Supporting this, studies have shown that American fathers tend to report lower self-regulation scores for their children’s eating behaviors compared to mothers [47]. To address these issues, healthcare professionals could advise parents of young children to stock their homes with healthier food options and less calorie-dense snacks [48]. Policymakers could incentivize and support parents to purchase healthier foods by stocking supermarkets located in neighborhoods with young families with fresh produce and healthier food options [49]. Future research should include validated measures from both parents to offer a more holistic understanding of parental feeding practices and their effects on children’s eating behaviors.

Pressure to eat and encouraging child control were positively associated with child food avoidance behaviors among young children. Similarly, parental utilization of pressure to eat was associated with a higher risk for child obesity in Asian studies conducted in Malaysia and Thailand [50,51,52]. However, pressuring young children to eat during mealtimes was found to be detrimental to children’s ability to self-regulate their food intake according to their cues of hunger and satiety, hence resulting in the child’s development of obesogenic behaviors [53,54]. Additionally, these disinhibited eating behaviors developed from parental pressure had enduring effects into adulthood [55,56]. Excessive support for child autonomy over their diet, coupled with minimal structure in mealtimes during early and middle childhood, is associated with greater food fussiness in children between one and seven years of age [57,58] and linked with poor dietary intake and an increased risk of childhood obesity [59]. At the other end of the spectrum, parental dieting behaviors and restrictive eating have also been found to impact the risk of eating disorders among adolescents [60]. Healthcare professionals should advise parents of young children to set healthy limits with their children through guided dietary choices, where parents give a few developmentally appropriate food choices at a set regular mealtime and allow the child to choose from the available food and determine their portion size through self-serving, to support the development of self-regulation in eating among children from a younger age [61]. At the same time, parents should also be encouraged to role-model healthy eating behaviors that are neither obesogenic nor overly restrictive.

Paternal use of food as a reward was found to be positively associated with food approach behaviors in school-aged children. Using food to soothe or regulate their children’s emotions was positively associated with food avoidance in school-aged children. At school-going age, children are often rewarded for good behaviors with unhealthy sweets and snacks. However, health guidelines from the American Academy of Pediatrics, the Academy of Nutrition and Dietetics, and the American Academy of Child and Adolescent Psychiatry have unanimously discouraged the use of food as a reward [62]. A study among British mothers also reported similar findings, where use of food for behavior and emotional regulation in children was associated with emotional overeating and undereating, respectively [63]. This is because such behavioral and emotional feeding practices encourage the child to have an increased preference for the reward food by reinforcing its desirability through physiological changes to neurological reward systems in their developing brains and creating an emotional link between such foods and achievements or self-soothing [64]. Additionally, cultural beliefs play a significant role in shaping parental feeding practices, and this is particularly evident in Asian families, where offering food as a reward for good behavior or using it to soothe emotional distress may be perceived as a loving gesture rather than a potentially harmful practice [65]. This cultural norm can inadvertently reinforce the use of food for behavior and emotional regulation, exacerbating the associations between food and emotional well-being. Healthcare professionals should raise awareness among parents about the unintended consequences of using food as a reward or for emotional regulation and provide alternatives, such as verbal praise, family activities, or non-food rewards like books or stickers. Schools also play an important role in reducing the use of unhealthy food-based rewards in classrooms. Future qualitative research could explore how Asian parents perceive the use of food as a reward or emotional regulator and how their cultural beliefs influence these practices.

Paternal practices of restricting a child’s diet to control their weight gain were positively associated with food approach behaviors and negatively associated with child food avoidance behaviors among school-aged children. Parental restriction during infancy to elementary school age is associated with child preference for restricted food items, excessive eating, and increased risk for childhood obesity [66]. This paradoxical effect may stem from overt parental control disrupting a child’s ability to self-regulate food intake [67]. Fathers could also be less inclined to restrict their child’s diet if they already perceive their child to have picky eating behaviors. Similar findings have also been described in another American study on maternal restrictive feeding practices [56]. Healthcare professionals should guide parents in adopting balanced approaches to weight management that focus on promoting healthy eating habits rather than strictly controlling or restricting certain foods, to foster positive food environments which support the development of healthy self-regulation in children. Further research should investigate the psychological mechanisms that drive restrictive feeding practices, particularly how parents’ perceptions of their child’s weight and eating behaviors influence their decision to restrict certain foods.

The results indicate that several factors, including fathers’ monitoring of their adolescents’ intake of unhealthy food and growing up in a low-income two-children Chinese household, are positively associated with food approach behaviors among adolescents. As adolescents develop increasing autonomy over time, parents are likely to use more covert approaches to control, such as monitoring their adolescents’ intake of sweets, snacks, and high-fat foods [68]. Likewise, adolescents who perceived higher levels of parental monitoring were also more likely to over-consume sugar-sweetened beverages in a recent Australian study [69]. When adolescents perceive their parents to engage in controlling feeding practices, they are encouraged to engage in secretive food-hiding behaviors, where they consume sugary beverages when they are away from their parents [70]. Clinicians should advise parents on the negative effects of overly controlling feeding practices, emphasizing the importance of fostering an open and supportive environment around food choices. Additionally, adolescents in low-income households have been shown to have a disproportionately higher prevalence of non-clinical binge eating, where they periodically consume large amounts of food with a sense of loss of control [71,72]. Multiple factors, such as food insecurity, trauma, poor nutritional knowledge, and weight stigmatization, could contribute to the manifestation of disordered eating cognitions among low-income adolescents [73,74]. Future research could explore the specific causal pathways and interactions between risk factors that lead to the development of binge eating behaviors among low-income adolescents, specifically examining socioeconomic factors, community resources, and stigma within the Asian context. Healthcare professionals should incorporate routine screening for unhealthy eating habits, especially among low-income adolescents, and raise awareness among low-income parents regarding their adolescents’ increased risk for unhealthy eating behaviors. A British study found that adolescents with siblings tend to eat faster than those without siblings, likely due to distractions during mealtime which prevent them from recognizing their internal satiety cues [75]. This can lead to the consumption of more food, even after they are full. Reciprocal peer influence among siblings may also prompt adolescents to eat unhealthy foods even in the absence of hunger, simply because they see their siblings eating [76]. Hence, healthcare professionals should take into consideration the presence of siblings at home when giving nutritional advice to parents. Training adolescents to eat slower and pay closer attention to their own internal hunger and satiety cues, rather than distractions in their external environment, could help enhance adolescents’ food responsiveness.

Fathers’ use of food to regulate their adolescents’ emotions, along with restricting their diet to limit the intake of unhealthy foods, was positively associated with food avoidance behaviors among adolescents. Using energy-dense foods and drinks for emotional comfort is strongly linked to both emotional overeating and undereating in adolescents [77]. High levels of stress and the internalization of negative emotions, more often encountered during adolescence, can result in adolescents having inhibited appetites and eating less [78]. Parental restriction can cause adolescents to feel shame associated with body image whilst ignoring their own internal hunger and satiety cues, predisposing them to maladaptive and disordered eating behaviors [79,80]. Healthcare professionals should raise parental awareness about the potential negative consequences of using restrictive feeding practices. Workshops that teach parents how to openly communicate their concerns regarding their adolescents’ health, without creating feelings of shame and guilt, could redirect parents to use more responsive and developmentally appropriate parenting practices [81]. Future qualitative research could also explore adolescents’ perspectives on their parents’ feeding practices, as understanding how adolescents perceive and respond to these practices could provide deeper insights into why certain approaches lead to food avoidance behaviors. Future interventional research could use biopsychosocial models to better understand how parental feeding practices, emotional regulation, and stress contribute to eating behaviors in adolescents [82,83]. These models could help identify complex interactions and inform more holistic treatment approaches.

Interestingly, adolescents whose fathers are employed full-time were reported by fathers to be less likely to exhibit food-avoidant behaviors, possibly due to less parental surveillance and control over their dietary patterns. In contrast, controlling and restrictive parenting practices have been widely documented to predict the manifestation of undereating behaviors among adolescents, as a coping mechanism for the perceived limitations on their autonomy and the resulting internal emotional turmoil [84]. Healthcare professionals should encourage fathers to continue being actively involved in various aspects of their adolescents’ nutrition, even when full-time employment limits their time at home. By offering practical strategies which allow fathers to reinforce healthy eating behaviors during the time they spend with their children—while also supporting their adolescents’ growing independence—healthcare providers can help ensure that fathers remain actively involved in fostering healthy eating habits throughout adolescence.

### Strengths and Limitations

Firstly, the participants for this study were recruited from a general pediatric outpatient clinic, which may have potentially introduced a selection bias. For example, as consultation fees at the study site were higher than the primary clinics situated within the community (polyclinics), there was a selection bias towards fathers from a higher socioeconomic status. More than half of the fathers in this sample reported a high household income (more than SGD 10,000), which is above the median household income in Singapore [85]. That said, the majority of individuals attending the general pediatric clinic were healthy patients who went to the clinic for immunizations, developmental assessments, or acute intercurrent ailments, without significant underlying chronic disease. The findings of this study would therefore still be relevant and applicable to the general Singaporean community. Future studies should recruit from multiple sites and employ diverse recruitment strategies to obtain a more representative sample. Secondly, the sample size of the study limited the analysis due to the reduced statistical power, especially among fathers of adolescents. Fathers who participated in this study were more likely to be more involved and conscious about their children’s eating behaviors, resulting in selection bias. Future research may consider recruiting mother–father dyads from the public to examine differences in involvement and perspectives on their feeding practices and triangulate the data to reduce self-reporting bias. Thirdly, the majority of the fathers in this study were Chinese, English-speaking, and highly educated, so there could be an over-representation of these fathers, resulting in limited generalizability of the results. Lastly, findings related to the subscales of “Involvement” and “Teaching about Nutrition” should be interpreted with caution due to the lower internal consistency values. Nevertheless, this is the first study to explore paternal involvement in influencing children’s eating behaviors in the multiracial Singaporean context. Understanding how fathers’ feeding practices can modify children’s eating behaviors can help lay the groundwork for more interventions to encourage paternal involvement and co-parenting to attain nutritional outcomes in children.

## 5. Conclusions

In conclusion, this study provides valuable insights into the paternal feeding practices prevalent among Singaporean fathers and their relationships with child eating behaviors. Among young children, the availability of healthy food was positively associated with food approach behaviors, while pressuring and child control were linked to increased food avoidance. Among school-aged children, the use of food as a reward and for regulating emotions was positively associated with food approach and food avoidance, respectively. Restriction for weight control was linked to food approach and avoidant behaviors. Among adolescents, monitoring their diet and certain socioeconomic conditions also influenced eating behaviors. Paternal feeding practices, which are potentially modifiable factors, could aid in efforts to reduce childhood obesity and help children develop healthy relationships with food from a young age. The findings from this study demonstrate the need for further in-depth research to explore other related constructs, such as nutritional knowledge and attitudes towards responsive feeding, the home food environment, and the complex interplay between parenting and family dynamics, sociocultural factors, and the child’s appetitive traits. Future research and interventions should take into consideration the cultural and socioeconomic factors that may influence paternal feeding practices through a participatory action design, with diverse samples of parents which are reflective of the target population.

## Figures and Tables

**Table 1:** Demographic characteristics of fathers and their children (*n* = 114).

Variables	Mean (SD)	*N*	%
Age	44.9 (6.17)		
Body mass index	25.8 (3.62)		
Citizenship			
Singapore citizen		91	79.8
Permanent resident		23	20.2
Ethnicity			
Chinese		80	70.2
Malay		8	7
Indian		16	14.0
Others (Caucasian, Burmese, Filipino)		10	8.8
Highest education level			
Secondary and below		5	4.4
Diploma and below		22	19.3
Tertiary and above		87	76.3
Partner’s highest education level			
Secondary and below		5	4.5
Diploma and below		17	15.5
Tertiary and above		92	80.0
Employment status			
Self-employed		14	12.3
Full-time		98	86.0
Part-time		1	0.9
Retired		1	0.9
Working hours per day	8.86 (1.55)		
Average household income per month			
<SGD 1000		1	0.9
SGD 1000–SGD 2999		1	0.9
SGD 3000–SGD 5999		19	16.7
SGD 6000–SGD 9999		24	21.1
>SGD 10,000		69	60.5
Number of children			
1		17	14.9
2		69	60.5
3 or more		28	24.6
Age of child (patient at the clinic)	8.64 (4.28)		
Sex of child (patient at the clinic)			
Male		68	59.6
Female		46	40.4
BMI Z-score (BAZ)	−0.10 (1.56)		

**Table 2:** General linear model of the association between paternal feeding practices and food approach or food-avoidant eating behaviors of their young children (0–6 years old) (*N* = 41).

Variables	β	*t*-Value	*p*-Value	95% CI for β
*Food approach*				
Environment	1.49	1.85	0.07	−0.14 to 3.13
Fathers’ working hours	−1.00	−2.29	0.02	−1.89 to −0.12
*Food avoidance*				
Pressure to eat	2.03	5.44	<0.001	1.28 to 2.80
Child control	0.733	2.23	0.032	0.07 to 1.40
Child gender				
Male	−3.49	−1.58	0.121	−7.95 to 0.96
Female	Reference			

**Table 3:** General linear model of the association between paternal feeding practices and food approach or food-avoidant eating behaviors of their school-aged children (7–12 years old) (*N* = 48).

Variables	β	*t*-Value	*p*-Value	95% CI for β
*Food approach*				
Child BMI z-score	0.86	1.16	0.254	−0.64 to 2.35
Restriction for weight control	0.39	2.62	0.012	0.09 to 0.69
Food as reward	0.73	2.10	0.042	0.03 to 1.43
Monitoring	−0.47	−1.50	0.142	−1.09 to 0.16
*Food avoidance*				
Emotional regulation	2.14	2.79	0.008	0.60 to 3.69
Restriction for weight control	−0.56	−2.57	0.013	−0.99 to −0.12

**Table 4:** General linear model of the association between paternal feeding practices and food approach or food-avoidant eating behaviors of their adolescents (13–18 years old) (*N* = 25).

CFPQ Subscales	β	*t*-Value	*p*-Value	95% CI for β
*Food approach*				
Monitoring	0.99	3.60	0.002	0.414 to 1.56
Ethnicity		5.36	0.020	
Chinese	5.70	2.32	0.032	0.545 to 10.8
Non-Chinese *				
Number of children		4.98	0.124	
One child	8.31	2.32	0.032	0.816 to 15.8
Two children	5.81	2.58	0.019	1.08 to 10.5
Three or more children *				
Monthly household income		6.28	0.022	
Less than SGD 6000	5.91	2.51	0.022	0.972 to 10.8
SGD 6000 and above *				
*Food avoidance*				
Emotional regulation	1.95	2.11	0.048	0.021 to 3.88
Restriction for health	1.90	3.85	<0.001	0.868 to 2.92
Employment status		9.91	0.010	
Employed full-time	−8.29	−2.85	0.010	−14.4 to −2.22
Not employed full-time *				

Reference categories were denoted with an asterisk (*).

## Data Availability

The original contributions presented in this study are included in the article/Supplementary Materials. Further inquiries can be directed to the corresponding author.

## Supplementary Materials

The following supporting information can be downloaded at: https://www.mdpi.com/article/10.3390/nu17050860/s1: Table S1: STROBE Checklist; Table S2: Child Feeding Practices Questionnaire and Child Eating Behavior Questionnaire Scores across three age groups; Table S3: Variation in child feeding practices among fathers across sociodemographic factors; and Table S4: Variation in child feeding practices among fathers across continuous variables.
