# Supplementary material for: Redefining Roles—Fathers Play a Crucial Role in Shaping Children’s Healthy Eating Behaviors: Cross-Sectional Observational Study
Source: Nutrients. 2025 Feb 28;17(5):860. doi: 10.3390/nu17050860 (PMC11901718; doi:10.3390/nu17050860)
Supplement: Supplementary file 1 [file nutrients-17-00860-s001.zip › nutrients-3480581-supplementary.pdf]

SUPPLEMENTARY FILE

Table S1: STROBE Checklist

STROBE Statement—Checklist of items that should be included in reports of ***cross-sectional studies***

	Item No	Recommendation	Page No
**Title and abstract**	1	(*a*) Indicate the study's design with a commonly used term in the title or the abstract	1
		(*b*) Provide in the abstract an informative and balanced summary of what was done and what was found	1
**Introduction**			
Background/rationale	2	Explain the scientific background and rationale for the investigation being reported	1-2
Objectives	3	State specific objectives, including any prespecified hypotheses	2
**Methods**			
Study design	4	Present key elements of study design early in the paper	2
Setting	5	Describe the setting, locations, and relevant dates, including periods of recruitment, exposure, follow-up, and data collection	2
Participants	6	(*a*) Give the eligibility criteria, and the sources and methods of selection of participants	2
Variables	7	Clearly define all outcomes, exposures, predictors, potential confounders, and effect modifiers. Give diagnostic criteria, if applicable	3
Data sources/ measurement	8*	For each variable of interest, give sources of data and details of methods of assessment (measurement). Describe comparability of assessment methods if there is more than one group	3
Bias	9	Describe any efforts to address potential sources of bias	3-4
Study size	10	Explain how the study size was arrived at	2
Quantitative variables	11	Explain how quantitative variables were handled in the analyses. If applicable, describe which groupings were chosen and why	3-4
Statistical methods	12	(*a*) Describe all statistical methods, including those used to control for confounding	3-4
		(*b*) Describe any methods used to examine subgroups and interactions	3-4
		(*c*) Explain how missing data were addressed	NA
		(*d*) If applicable, describe analytical methods taking account of sampling strategy	NA

		(*e*) Describe any sensitivity analyses	NA
**Results**			
Participants	13*	(a) Report numbers of individuals at each stage of study—eg numbers potentially eligible, examined for eligibility, confirmed eligible, included in the study, completing follow-up, and analysed	4-5
		(b) Give reasons for non-participation at each stage	NA
		(c) Consider use of a flow diagram	NA
Descriptive data	14*	(a) Give characteristics of study participants (eg demographic, clinical, social) and information on exposures and potential confounders	4-5, Table 1
		(b) Indicate number of participants with missing data for each variable of interest	NA
Outcome data	15*	Report numbers of outcome events or summary measures	5
Main results	16	(*a*) Give unadjusted estimates and, if applicable, confounder-adjusted estimates and their precision (eg, 95% confidence interval). Make clear which confounders were adjusted for and why they were included	5-7
		(*b*) Report category boundaries when continuous variables were categorized	5-7
		(*c*) If relevant, consider translating estimates of relative risk into absolute risk for a meaningful time period	NA
Other analyses	17	Report other analyses done—eg analyses of subgroups and interactions, and sensitivity analyses	NA
**Discussion**			
Key results	18	Summarise key results with reference to study objectives	7
Limitations	19	Discuss limitations of the study, taking into account sources of potential bias or imprecision. Discuss both direction and magnitude of any potential bias	10
Interpretation	20	Give a cautious overall interpretation of results considering objectives, limitations, multiplicity of analyses, results from similar studies, and other relevant evidence	7-10
Generalisability	21	Discuss the generalisability (external validity) of the study results	7-10
**Other information**			
Funding	22	Give the source of funding and the role of the funders for the present study and, if applicable, for the original study on which the present article is based	11

*Give information separately for exposed and unexposed groups.

Note: An Explanation and Elaboration article discusses each checklist item and gives methodological background and published examples of transparent reporting. The STROBE checklist is best used in conjunction with this article (freely available on the Web sites of PLoS Medicine at http://www.plosmedicine.org/, Annals of Internal Medicine at http://www.annals.org/, and Epidemiology at http://www.epidem.com/). Information on the STROBE Initiative is available at www.strobe-statement.org.

Table S2: Child Feeding Practices Questionnaire and Child Eating Behavior Questionnaire Scores across three age groups

Domains	Mean Score (SD)		
	**0-6 years old**	**7-12 years old**	**13-18 years old**
Child Feeding Practices Questionnaire			
Child Control	2.66 (3.32)	2.82 (3.26)	2.72 (2.50)
Emotion Regulation	1.98 (2.18)	1.93 (1.96)	1.83 (1.53)
Balance and Variation	*excluded from analysis due to low internal consistency		
Environment	3.77 (2.54)	3.65 (2.62)	3.95 (2.74)
Food as Reward	2.90 (2.99)	2.53 (2.88)	2.32 (3.07)
Involvement	3.50 (2.28)	3.43 (2.21)	3.80 (2.71)
Modeling	3.70 (3.36)	3.70 (3.57)	3.95 (3.17)
Monitoring	3.75 (2.95)	3.53 (3.46)	3.38 (4.31)
Pressure	3.53 (2.99)	3.10 (3.75)	3.03 (2.99)
Restriction for health	3.55 (3.15)	3.63 (3.17)	3.63 (3.86)
Restriction for weight control	2.61 (4.92)	2.61 (6.91)	2.93 (7.31)
Teaching	3.93 (2.19)	4.13 (1.98)	4.30 (2.00)
Child Eating Behaviors Questionnaire			
Food responsiveness	3.23 (3.30)	2.98 (3.55)	3.18 (3.60)
Enjoyment of food	3.53 (2.62)	3.57 (2.71)	3.65 (3.08)
Emotional overeating	1.88 (2.39)	1.90 (2.44)	1.92 (2.54)
Desire to drink	2.77 (2.58)	2.52 (2.61)	2.83 (2.08)

**Total food approach**	**42.8 (7.51)**	**41.3 (7.73)**	**43.5 (7.22)**
Satiety responsiveness	2.98 (3.04)	2.88 (3.30)	2.78 (2.63)
Slowness in eating	3.30 (2.96)	2.85 (3.87)	2.65 (2.71)
Food Fussiness	2.54 (3.60)	2.49 (4.86)	2.43 (5.45)
Emotional undereating	3.15 (3.00)	2.85 (2.95)	2.75 (3.31)
**Total food avoidance**	**58.5 (9.92)**	**54.5 (11.6)**	**52.5 (9.50)**

Table S3: Variation in child feeding practices among fathers across sociodemographic factors

Variables	Mean Difference (95% CI)	t/F-value	p-value
**Young children aged 0-6 years old**			
	Emotion Regulation		
Education Level	-1.81 (-3.57 to -0.04)	-2.07	0.045
	Pressure to Eat		
Education Level	-3.02 (-5.36 to -0.67)	-2.60	0.013
**School-aged children aged 7-12 years old**			
	Food as reward		
Nationality	2.11 (0.39 to 3.82)	2.47	0.017
Employment Status	-2.33 (-4.63 to -0.03)	-2.03	0.047
	Modelling		
Nationality	-2.01 (-3.85 to -0.17)	-2.21	0.033
	Monitoring		
Employment Status	3.52 (0.83 to 6.20)	2.63	0.011
Monthly Household Income	***Emotion Regulation***	3.05	0.05
Less than S$6,000	Reference		
S$6,000 to S$9,999	2.39 (0.04 to 4.75)		0.046
More than S$10,000	1.39 (-0.51 to 3.28)		
No. of children	***Restriction for Weight Control***	4.07	0.024
1	-4.07 (-11.6 to 3.42)		0.682
2	-6.36 (-11.8 to -0.95)		0.018
3 or more	Reference		

**Adolescents aged 13-18 years old**			
	Child Control		
Nationality	2.80 (0.18 to 5.41)	2.21	0.037
	Pressure		
Partner's Education Level	-3.49 (-5.73 to -1.23)	-3.20	0.004
Monthly Household Income		3.32	0.055
Less than S$6,000	-4.04 (-8.70 to 0.61)		0.097
S$6,000 to S$9,999	-4.45 (-8.82 to -0.08)		0.047
More than S$10,000	Reference		
	Emotion Regulation		
Adolescent's Gender	-1.51 (-2.69 to -0.33)	-2.64	0.015
Monthly Household Income		4.56	0.022
Less than S$6,000	Reference		
S$6,000 to S$9,999	1.02 (-0.48 to 2.51)		0.225
More than S$10,000	2.71 (0.42 to 4.99)		0.018
	Involvement		
Adolescent's Gender	-2.44 (-4.58 to -0.30)	-2.36	0.027
Ethnicity	***Food as Reward***	4.29	0.016
Chinese	Reference		
Malay	-5.81 (-11.2 to -0.40)		0.032
Indian	-2.56 (-6.59 to 1.47)		0.314
Others	-3.31 (-7.85 to 1.23)		0.208
No. of children	***Environment***	3.76	0.039
1	4.47 (0.38 to 8.56)		0.031
2	1.05 (-1.61 to 3.71)		0.590

3 or more	Reference		
No. of children	***Modelling***	4.53	0.023
1	Reference		
2	-4.92 (-9.44 to -0.40)		0.031
3 or more	-5.37 (-9.98 to -0.76)		0.021
No. of children	***Monitoring***	3.25	0.058
1	Reference		
2	-6.42 (-12.8 to 0.00)		0.050
3 or more	-4.43 (-10.9 to 2.11)		0.227

Table S4: Variation in child feeding practices among fathers across continuous variables.

Variables	Feeding Practice	r (p-value)	95% CI
**Young children aged 0-6 years old**			
Child BMI z-score	Emotion Regulation	-0.372 (0.02)	-0.61 to -0.07
	Environment	0.366 (0.02)	0.07 to 0.61
	Food as Reward	-0.393 (0.01)	-0.63 to -0.10
	Involvement	0.353 (0.02)	0.05 to 0.60
	Pressure	-0.398 (0.01)	-0.63 to -0.10
Fathers' Working Hours	Environment	-0.314 (0.05)	-0.61 to -0.05
**School-aged children aged 7-12 years old**			
Child BMI z-score	Environment	-0.383 (0.01)	-0.60 to -1.11
	Modelling	-0.40 (0.01)	-0.61 to -0.13
	Monitoring	-0.34 (0.02)	-0.57 to -0.06
Fathers' BMI	Pressure	-0.291 (0.04)	-0.53 to -0.01
Fathers' Working Hours	Involvement	0.318 (0.03)	0.04 to 0.55
**Adolescents aged 13-18 years old**			
Child BMI z-score	Pressure	-0.486 (0.01)	-0.74 to -0.11
	Restriction for Weight Control	0.398 (0.05)	0.00 to 0.69
Fathers' Age	Restriction for Weight Control	0.472 (0.02)	0.09 to 0.73
Father's BMI	Environment	-5.21 (0.01)	-0.76 to -0.16
Father's Working Hours	Pressure	-0.45 (0.02)	-0.71 to -0.06
